# Evaluation of Thyroid Nodules in Patients With Fine-Needle Aspiration Biopsy

**DOI:** 10.7759/cureus.44569

**Published:** 2023-09-02

**Authors:** Ebru Turkkan, Yusuf Uzum

**Affiliations:** 1 Internal Medicine, Katip Celebi University, Ataturk Training and Research Hospital, Izmir, TUR

**Keywords:** thyroid disorder, thyroid, biopsy, thyroidectomy, thyroid nodules, fine-needle aspiration biopsy

## Abstract

Background: The incidence of thyroid nodules has increased in the last 50 years due to the widespread use of imaging methods and incidental detection of small thyroid nodules. Thyroid fine-needle aspiration biopsy (FNAB) is the most accurate, reliable, and cost-effective test to evaluate thyroid nodules.

Aim: In this research, we aimed to elucidate thyroid fine-needle aspiration cytology (FNAC) to understand how suspicious cases predict malignancy.

Materials and methods: Within this research’s scope, 411 patients over 16 years old who were evaluated in Izmir Katip Celebi University, Ataturk Training and Research Hospital Internal Medicine (Izmir, Turkey) outpatient clinic for thyroid nodules between 2018 and 2022 and underwent thyroid FNAC followed by thyroid surgery were analyzed retrospectively. The age, gender, thyroid FNAC, operation type, and histopathology of all the patients were reviewed. Individuals with a history of head and neck cancer were excluded from the analysis.

Results: No statistically significant relationship between the pathology results and demographic characteristics was found. A statistically significant correlation existed between the pathology and FNAB results (p<0.05)*.* Although 84.5% of the patients were diagnosed as benign, 14.7% as suspicious, and 0.8% as malignant in FNAC, all of these cases were diagnosed as benign in final histopathology results. Similarly, 21.9% of the patients were diagnosed as benign, 58.8% as suspicious, and 19.4% as malignant in FNAC and all of these cases were diagnosed as malignant in final histopathology results. A correlation was determined between the two measurements (Cohen’s kappa (κ)=0.557; p<0.001). The test’s sensitivity was 47%, and the specificity was 99.1%. According to the FNAC results, the rate of being diagnosed with malignancy (positive predictive value (PPV)) was 93.9%, and the rate of being diagnosed as benign (negative predictive value (NPV)) was 85.8% for the individuals initially diagnosed as benign.

Conclusion: Although FNAB remains the most important diagnostic tool to identify benign cases with a high accuracy rate, the operation decision is not clear in suspicious atypia of undetermined significance/follicular lesions of undetermined significance (AUS/FLUS) cytology findings. In conclusion, this study highlights the importance of FNA results and helps in surgical decision-making by emphasizing that the possibility of malignancy in the post-operative final histopathology report is higher, especially in the presence of suspicious FNAC results.

## Introduction

Thyroid nodules are clinically common endocrine pathologies that most frequently require surgical intervention. The incidence of thyroid nodules has increased in the last 30 years owing to the widespread use of imaging methods and the incidental detection of small thyroid nodules [1,2]. Thyroid nodules are found in almost half of the population, and their incidence increases with age. Incidental thyroid nodules are seen in more than 20% of chest CT scans and in almost 20% of CT and MRI scans. The increase in thyroid nodule detection rates over the last 30 years may be related to these incidental cases. Thyroid fine-needle aspiration biopsy (FNAB) is the most accurate, reliable, and cost-effective test to evaluate thyroid nodules. FNAB has a long history of clinical practice in diagnosing thyroid nodules. The diagnostic criteria allow follow-up and treatment options after FNAB to be clarified. As the diagnosis of thyroid nodules increases, the frequency of diagnosis of thyroid carcinoma also increases. The primary goal is differentiating between FNAB and pre-operative, benign, or malignant nodules [2].

Thyroid nodules are among the most common endocrine pathologies. In Western countries, the rate of thyroid nodules ranges from 3% to 8%. In Turkey, the prevalence of thyroid nodules was reported as 2-6% with palpation examination and 18% with ultrasonography (USG). Thyroid cancers constitute 1% of all malignant neoplasms and are seen three to four times more frequently in women than in men [3].

In routine clinical practice for thyroid nodules, important diagnostic information is obtained by thyroid function tests, scintigraphy, and USG. However, the distinction between benign and malignant lesions cannot be conducted precisely with these tests [3]. When thyroid function tests are analyzed in thyroid nodules, thyroid hormones may be elevated. Not every thyroid hormone elevation indicates the presence of a thyroid nodule, and thyroid hormones may not be elevated in every thyroid nodule. Meanwhile, FNAB is accepted as the most reliable diagnostic method for distinguishing between benign and malignant thyroid nodules. Recently, with the USG-guided FNAB application, it has become possible to aspirate deeper nodules. To date, FNAB remains the primary tool for assessing the nature of thyroid nodules. A decrease in thyroidectomies and an increase in the malignancy rate in operated thyroid nodules were detected via FNAB. However, more than 50% of operated thyroid nodules are reported as benign [2,4].

The malignant potential in asymptomatic individuals highlights the clinical importance of investigating thyroid nodules. Increased diagnostic accuracy has improved the non-operative follow-up of benign thyroid lesions. Appropriate management of thyroid papillary microcarcinoma cases and large nodules is debatable [5]. It remains a concern for clinicians treating patients with thyroid nodules with a false-negative FNAB result. False-negative FNAB cytology causes patients to be diagnosed at a later stage and delayed treatment. Studies suggest that if there is more than one thyroid nodule larger than 1 cm, especially in male patients, a biopsy should be performed for possible thyroid carcinoma from other nodules other than the nodule with malignant potential [6]. Moreover, the American Thyroid Association (ATA) guidelines recommend ultrasound-guided fine-needle aspiration cytology (US-FNAC) for nodules >1 cm in size [6]. USG findings suggestive of malignancy include solid and hypoechoic nodules, microcalcifications, irregular nodule borders, absence of halo, nodule length longer than width, and increased nodule vascularity on Doppler ultrasound.

It has been reported that FNAB may generate false negativity in patients with multinodular goiter (MNG), and the reliability of FNAB is quite high in solitary nodules. However, there is controversy regarding the accuracy of FNAB for nodules smaller than 1 cm or larger than 4 cm. USG-guided FNAB has a low complication risk in diagnosing malignant thyroid nodules [7].

In this research, we aimed to elucidate thyroid FNAC to understand how suspicious cases predict malignancy. In the previous literature, it has been reported that a preoperative diagnosis of malignancy will enable a single-stage surgery.

## Materials and methods

Within the scope of this research, 411 patients who applied to Izmir Katip Celebi University, Ataturk Training and Research Hospital Internal Medicine (Izmir, Turkey) outpatient clinic with a thyroid nodule between 2018 and 2022 and underwent thyroid FNAC followed by thyroid surgery were analyzed retrospectively. The age, gender, thyroid FNA cytology, operation type, and histopathology of all the patients were evaluated. 

In our hospital, all ultrasonographic examinations for the thyroid pathologies and simultaneous thyroid FNAB procedures are performed by a single specialized operator.

To make standardization of diagnostic terminology, morphologic criteria, and risk of malignancy for reporting of thyroid FNA, in 2007, the National Cancer Institute (NCI) proposed a six-tier system and named it the Bethesda System for Reporting Thyroid Cytopathology (BSRTC). The BSRTC that was developed to refine FNAC definitions and improve clinical management was used for cytological diagnosis. The risks of malignancy for the categories of nondiagnostic (I), benign (II), atypia of undetermined significance (AUS)/follicular lesion of undetermined significance (FLUS) (III), follicular neoplasm (FN)/suspicious for follicular neoplasm (SFN) (IV), suspicious for malignancy (SM) (V), and malignant (VI) were 1-4%, 0-3%, 5-15%, 15-30%, 60-75%, and 97-99%, respectively.

The operation type is classified as total thyroidectomy, partial thyroidectomy, hemithyroidectomy, and subtotal thyroidectomy. Histopathological evaluation was classified as benign and malignant, and their subtypes were also classified.

In particular, cases with suspicious FNA cytology results according to the BSRTC classification were specified, and their histopathological correlation was examined.

All procedures followed were in accordance with the ethical standards of the responsible committee on human experimentation (institutional and national) and with the Helsinki Declaration of 1975, as revised in 2008. Ethics committee approval has been granted from our institution on April 21, 2022, with protocol number 0169.

Inclusion criteria

Patients ≥16 years old who applied to the Izmir Katip Celebi University, Ataturk Training and Research Hospital Internal Medicine outpatient clinic with a thyroid nodule between 2018 and 2022 were included.

Exclusion criteria

Patients with missing medical data, individuals with a history of head and neck cancer, and those who underwent operation for thyroid or parathyroid pathology were excluded from the analysis.

Statistical analysis

Analyses were performed using IBM SPSS Statistics for Windows, version 23 (released 2015; IBM Corp., Armonk, New York, United States). The characteristics of patients, i.e., *n* (percent) and mean ± standard deviation for categorical and continuous variables, respectively, were compared among treatment groups using Pearson chi-square/Fisher’s exact test or independent sample t-tests, as appropriate. Cohen’s kappa (κ) coefficient was used to evaluate the conformity between the FNA and pathology results. The P value was set at <0.05 for statistical significance.

## Results

A total of 411 patients who were admitted to our institution were enrolled in this retrospective analysis. A majority of the cases were female (n=353, 85.9%), and 14.1% (n=58) were male. The pathology results showed that adenomatous nodular hyperplasia was the most common disease in 28.2% (n=116), followed by papillary carcinoma in 28% (n=115), adenomatous nodule in 20% (n=82), follicular adenoma in 9.7% (n=40), follicular variant of papillary carcinoma in 5.1% (n=21), and follicular carcinoma in 3.2% (n=13). Hurtle-cell carcinoma, medullary carcinoma, nodular hyperplasia, metastasis, thyroiditis, and undifferentiated carcinoma were quite rare and less than 2%.

Regarding the type of surgery, total thyroidectomy was conducted in 79% (n=325), hemithyroidectomy in 14.4% (n=59), subtotal thyroidectomy in 3.4% (n=14), and one-side hemithyroidectomy and other-side partial thyroidectomy in 3.2% (n=13). The demographic and clinical data of the study patients are given in Table 1.

**Table 1:** Demographic and clinical data of the study patients FNA, fine-needle aspiration; FLUS, follicular lesion of undetermined significance

Characteristics (n=411)	n (%) or mean ± SD
Age (years)	52±14 (min: 17; max: 89)
Sex	
Female	353 (85.9)
Male	58 (14.1)
FNA	
Benign	247 (60.1)
Suspicious	131 (31.9)
Malignant	33 (8)
FNA specification	
FLUS	3 (0.7)
Benign	247 (60.1)
Malignant	33 (8)
Suspicious for follicular neoplasm	32 (7.8)
Suspicious for hurtle cell neoplasm	14 (3.4)
Suspicious for malignancy	82 (20)
Pathology	
Benign	251 (61.1)
Malignant	160 (38.9)
Final histopathology	
Adenomatous nodular hyperplasia	116 (28.2)
Adenomatous nodule	82 (20)
Follicular adenoma	40 (9.7)
Follicular carcinoma	13 (3.2)
Hurtle cell adenoma	8 (1.9)
Hurtle cell carcinoma	4 (1)
Medullary carcinoma	4 (1)
Metastasis	4 (1)
Nodular hyperplasia	2 (0.5)
Papillary carcinoma	115 (28)
Papillary carcinoma follicular variant	21 (5.1)
Thyroiditis	1 (0.2)
Undifferentiated carcinoma	1 (0.2)
Surgery type	
Total thyroidectomy	325 (79)
One-side hemithyroidectomy and other-side partial thyroidectomy	13 (3.2)
Subtotal thyroidectomy	14 (3.4)
Hemithyroidectomy	59 (14.4)

The distribution of demographic and FNAB results according to the patients' final histopathology results is denoted in Table 2. When the table was examined, no statistically significant relationship was found between the histopathology results and demographic characteristics (p>0.05). A statistically significant correlation existed between the pathology and FNAB results (p<0.05). Although 84.5% of the patients were diagnosed as benign, 14.7% as suspicious, and 0.8% as malignant in FNAC, all of these cases were diagnosed as benign in final histopathology results. Similarly, 21.9% of the patients were diagnosed as benign, 58.8% as suspicious, and 19.4% as malignant in FNAB and all of these cases were diagnosed as malignant in final histopathology results.

**Table 2:** Evaluation of the demographic and FNAC results with the histopathology results of the study patients FNA, fine-needle aspiration; FNAC, fine-needle aspiration cytotology

Variables	Pathology		
	Benign (n=251)	Malignant (n=160)	p-value
	n (%) or Mean ± SD	n (%) or Mean ± SD	
Age (years)	52±14	51±14	0.307
Sex			0.577
Female	218 (86.9)	135 (84.4)	
Male	33 (13.1)	25 (15.6)	
FNA			
Benign	212 (84.5)	35 (21.9)	<0.001
Suspicious	37 (14.7)	94 (58.8)	<0.001
Malignant	2 (0.8)	31 (19.4)	<0.001

The results of evaluating the concordance between the FNAB results of the patients and the final histopathology results are denoted in Table 3. When the table was examined, a general correlation was determined in the two measurements (κ: 0.557, p<0.001). The test’s sensitivity was 47%, and the specificity was 99.1%. According to the FNAB test, the rate of being diagnosed with malignancy (positive predictive value (PPV)) was 93.9%, and the rate of being diagnosed as benign (negative predictive value (NPV)) was 85.8 for the individuals diagnosed as benign.

**Table 3:** Conformity of the FNAC and histopathology results of the study patients PPV, positive predictive value; NPV, negative predictive value; κ, Cohen’s kappa coefficient; FNR, false-negative rate; FPR, false-positive rate.

	Pathology				n=280
FNAB	Malignant	Benign	Total	FNR	53.0%
Malignant	31	2	33	FPR	0.9%
Benign	35	212	247	Sensitivity	47.0%
Total	66	214	280	Specificity	99.1%
				PPV	93.9%
				NPV	85.8%
				Accuracy	86.8%
				k	0.557
				p-value	<0.001

## Discussion

FNAB is a very valuable method used in the evaluation of thyroid nodules, differentiation of benign and malignant, and selection of patients who require surgical treatment. The mean sensitivity in detecting thyroid cancers was 83% (65-98%), the specificity was 92% (72-100%), and the diagnostic accuracy was 95%. With the widespread use of FNAB in evaluating thyroid nodules, the number of patients undergoing surgery has decreased by 35-75%. The rate of thyroid carcinoma detected in surgery increased by two- to threefold. FNAB is a simple, inexpensive, well-tolerated diagnostic method that can be applied in outpatient settings, can be repeated when necessary, and has few side effects, such as hematoma [8]. Biopsy should be performed by experienced physicians who have received training on this subject. Experienced cytopathologists should evaluate FNAB samples. In the evaluation of large FNAB series, nodules were 70% (53-90%) benign, 4% (1-10%) malignant, and 10% (5-23%) suspicious or unclear (follicular or Hurthle cell tumor). On the contrary, 17% (15-20%) were reported as insufficient materials [9,10]. In this study, we have found a statistically significant correlation between the final histopathology and FNAB results. According to the cytology results, 84.5% of the patients' lesions were benign, 14.7% suspicious, and 0.8% malignant in the FNAB. Similarly, according to the final histopathology results, 21.9% of the patients' lesions initially diagnosed as malignant were benign, 58.8% suspicious, and 19.4% malignant in the FNAB.

For biopsy materials to be considered adequate, at least six cell populations containing 10-20 well-preserved follicular epithelial cells should be seen. An insufficient material is the most common cause of false-negative results [11]. In this case, FNAB should be repeated under US guidance. It has been shown that the rate of non-diagnostic results decreases from 3% to 15% if the biopsy is performed again with the help of US. It was determined that 50% of the nodules, persistently non-diagnostic as a result of FNAC, were malignant. Therefore, these patients should be given surgical treatment or followed closely [12].

In non-palpable, small (1-1.5 cm), cystic, and mixed nodules, FNAB should be performed under US guidance. In cystic and mixed nodules, the material should be taken from the cyst wall and the solid part of the nodüle [13]. Cystic fluid should be aspirated completely and sent to pathology. In the presence of multiple nodules (MNG), evaluating the nodules with US (Doppler) for malignancy indicators is recommended. However, thyroid USG is a complementary method in evaluating thyroid nodules, not an alternative to biopsy. For this reason, it is recommended to perform a separate biopsy from all accessible nodules in MNG, if possible [14].

While the FNAB of thyroid nodules can recognize papillary, medullary, and anaplastic carcinomas, follicular carcinoma cannot be diagnosed. The vascular or capsular invasion should be demonstrated to diagnose follicular carcinoma. These cannot be seen in an FNAB material, and the material is defined as “FN.” Nodules defined as FNs are in the suspicious group for malignancy [15]. Surgical treatment is recommended for these patients. It has been reported that 20-25% of the patients who were operated for FN have follicular carcinoma, and the remaining 75-80% result from benign pathology. To prevent these unnecessary operations, cytopathologists use immunocytochemical and genetic markers to differentiate benign from malignant nodules with cytology results suspicious of FN or malignancy [16]. While FNAB allows for the diagnosis of cancer or benign nodules in most patients, 20-30% of cytology specimens yield one of three types of uncertain cytological diagnosis (atypia/follicular lesion of insignificant significance, follicular neoplasia/suspect follicular neoplasia, or suspected malignancy) [17]. In a meta-analysis of 25,445 thyroid FNA samples reported from eight studies using the BSRTC, 9.6% of all the samples were AUS/FLUS, 10.1% were follicular neoplasia/follicular neoplasia suspected, and 2.7% were malignant. Their average cancer risk is 15.9%, 26.1%, and 75.2%, respectively. These cancer risks are not low enough to delay surgical treatment altogether. However, with the exception of cytology with suspected malignancy, the risks are not high enough to warrant a definitive indication for cancer surgery [18]. In conclusion, a typical diagnostic lobectomy performed on most of these patients will be an unnecessary surgery if the nodule proves benign after surgery, an inadequate surgery for some nodules identified as malignant after surgery.

Due to the low predictive value of FNAB in thyroid cancers, some argue that it should be used especially to define benign lesions. The reason is the inability to differentiate between follicular carcinoma and adenoma. As a result of the cytological examination, the condition is classified as benign, intermediate (follicular neoplasia or suspected follicular neoplasia), and malignant. Because carcinoma is diagnosed by looking at capsule and vessel invasion in FNs, the fact that this condition cannot be evaluated with FNAB causes these lesions to be evaluated among suspicious lesions [19]. Considering all these reasons, it raises the question of whether using positron emission tomography (PET) CT as an additional imaging method will have a diagnostic value, especially in patients with suspected follicular neoplasia preoperatively, apart from USG and FNAB [20].

This study evaluated the results of the contribution of the FNAB method as a diagnosis in the approach to thyroid cancers. It has been determined that FNAB helps clinicians decide based on the patient's history, physical examination, and noninvasive diagnostic methods rather than an obvious contribution to when and to what extent surgical intervention should be performed in thyroid nodules. Numerous articles have recommended FNAB as an important step in the approach to solid thyroid nodules. Identifying a malignant tumor before surgery makes it easier to plan for surgery [21].The sensitivity of FNAB in our study was 47%, and the specificity was 99.1%. According to the FNAB test, the rate of being diagnosed with malignancy was 93.9%, and the rate of being diagnosed as benign was 85.8%. The sensitivity and specificity of FNAB are at different rates in different studies. Gardiner et al. reported these rates as 65-91%; according to Hawkins, these rates were 86-95%; in Caplan et al., 91-99%; and in Burch et al., 80-73% [22,23,24,25]. The high specificity of thyroid FNAC easily distinguishes cases without diseases and reduces the possibility of unnecessary surgery. This is important both in terms of managing the patient's risk related to the surgical procedure and national health expenditures. 

The combination of FNAB results, ultrasound findings, molecular testing, and patient history and preference should all be carefully assessed together to determine the best course for surgical treatment. The most important limitation of our study is the retrospective nature of the study. Moreover, the number of cases participating in retrospectively designed studies as much as possible may increase the power of the study.

## Conclusions

Although FNAB remains the most important diagnostic tool to identify benign cases with a high accuracy rate, the operation decision is not clear in suspicious AUS/FLUS cytology findings. This study highlights the importance of FNA results and helps in surgical decision-making by emphasizing that the possibility of malignancy in the post-operative final histopathology report is higher, especially in the presence of suspicious FNA cytology results.

Finally, suspicious FNAC results should not be underestimated, the possibility of malignancy should always be kept in mind in these cases, and necessary plans for final diagnosis and treatment should be made quickly.
